# eIF4E binding protein 1 expression is associated with clinical survival outcomes in colorectal cancer

**DOI:** 10.18632/oncotarget.4483

**Published:** 2015-06-29

**Authors:** Min-Wu Chao, Li-Ting Wang, Chin-Yu Lai, Xiao-Ming Yang, Ya-Wen Cheng, Kuo-Hsiung Lee, Shiow-Lin Pan, Che-Ming Teng

**Affiliations:** ^1^ Pharmacological Institute, College of Medicine, National Taiwan University, Taipei, Taiwan; ^2^ Natural Products Research Laboratories, Eshelman School of Pharmacy, University of North Carolina at Chapel Hill, Chapel Hill, NC, USA; ^3^ Chinese Medicine Research and Development Center, China Medical University and Hospital, Taichung, Taiwan; ^4^ The Ph.D. Program for Cancer Biology and Drug Discovery, College of Medical Science and Technology, Taipei Medical University, Taipei, Taiwan; ^5^ Department of Pharmacology, College of Medicine, Taipei Medical University, Taipei, Taiwan

**Keywords:** colorectal cancer (CRC), eIF4E binding protein 1, hypoxia, prognosis, YXM110

## Abstract

eIF4E binding protein 1 (4E-BP1), is critical for cap-dependent and cap-independent translation. This study is the first to demonstrate that 4E-BP1 expression correlates with colorectal cancer (CRC) progression. Compared to its expression in normal colon epithelial cells, 4E-BP1 was upregulated in CRC cell lines and was detected in patient tumor tissues. Furthermore, high 4E-BP1 expression was statistically associated with poor prognosis. Hypoxia has been considered as an obstacle for cancer therapeutics. Our previous data showed that YXM110, a cryptopleurine derivative, exhibited anticancer activity via 4E-BP1 depletion. Here, we investigated whether YXM110 could inhibit protein synthesis under hypoxia. 4E-BP1 expression was notably decreased by YXM110 under hypoxic conditions, implying that cap-independent translation could be suppressed by YXM110. Moreover, YXM110 repressed hypoxia-inducible factor 1α (HIF-1α) expression, which resulted in decreased downstream vascular endothelial growth factor (VEGF) expression. These observations highlight 4E-BP1 as a useful biomarker and therapeutic target, indicating that YXM110 could be a potent CRC therapeutic drug.

## INTRODUCTION

Colorectal cancer (CRC) is the third most common cancer and the third leading cause of cancer related deaths worldwide [1]. Approximately 50–60% of diagnosed CRC patients will develop metastases. Most cases of CRC are sporadic, with only a small proportion related to heredity. It has been reported that some gene mutations increase the incidence of CRC, including those of *APC* (adenomatous polyposis coli), *KRAS*, *MLH1*, *MSH2*, *TP53*, and *EGFR* [2]. Currently, the cornerstone treatment for CRC is surgery for stage I cases, with adjuvant radiotherapy and systemic chemotherapy for stages II and III. In addition to traditional chemotherapy drugs (5-fluorouracil, irinotecan, and oxaliplatin), several targeted monoclonal antibodies such as bevacizumab, cetuximab, panitumumab, and capecitabine have been utilized in the clinic [3].

There has been an increased focus in the past few years on individualized therapy for cancer treatment. Traditional systemic chemotherapy often results in off-target toxicity in patients with a resulting loss in quality of life. Therefore, the development of prognostic and predictive markers would be advantageous for the development of targeted therapeutics. Recently, several valuable molecular markers have been discovered for CRC. Microsatellite instability (MSI), a kind of genome instability, results from a dysfunction in mismatch repair genes and often occurs in cancers. An evaluation of the effect of high MSI on patient survival in 5-FU treated and untreated CRC patients demonstrated that untreated patients with high MSI had improved overall survival compared to high MSI patients treated with 5-FU [4]. MSI, 18q loss of heterozygosity, p53, SMAD4, thymidylate synthase, KRAS, and BRAF genes have been shown to be important indicators of CRC pathogenesis. Other markers, such as the tumor suppressor, guanylyl cyclase 2 and the nucleotide excision repair gene, ERCC1 are also being considered as possible predictive markers [5], which emphasizes the importance of identifying relevant biomarkers for the design of targeted drugs.

Protein synthesis controls cell growth, cell size maintenance, proliferation, and the survival of cancer cells [6]. Translation phases include the rate-limiting step of initiation, the assembly of the elongation-competent 80S ribosome, and termination. Translation of mRNAs can occur in a cap-dependent and cap-independent manner. Cap-tagged mRNAs recruit eukaryotic initiation factors (eIFs), forming the eIF4F complex, composed of the following proteins: scaffolding protein eIF4G, RNA helicase eIF4A, and the cap-binding protein eIF4E [7]. eIFs work together to initiate the translation process. However, recent studies have indicated that cap-independent translation may contribute to cancer progression. Unlike cap-dependent translation, cap-independent translation involves access to mRNAs through an internal ribosome entry site (IRES) without cap binding. Under cellular stress such as in hypoxia, cap-independent mRNAs can be translated even when cap-dependent translation is decreased [8]. Cellular mRNAs that also contain IRES elements include hypoxia-inducible factor 1α (*HIF1A*), vascular endothelial growth factor (*VEGFA*), fibroblast growth factor 2 (*FGF2*), and *BCL2*, all of which utilize a dual mechanism of initiation (cap or IRES) [9, 10].

4E-BP1, the most common eukaryotic translation initiation factor of 4E binding proteins (4E-BPs) impacts cell proliferation, apoptosis, invasion, and metastasis. The hypophosphorylated active form of 4E-BP1, sequesters eIF4E and prevents it from binding to eIF4G, thus blocking the formation of the cap-dependent translation complex (eIF4F). 4E-BP1 also activates cap-independent translation by binding to eIF4E [11]. It has been shown that elevated levels of 4E-BP1 and eIF4G induces hypoxia mediated inhibition of cap-dependent mRNA translation and increases the translation of mRNAs containing IRES. This leads to angiogenesis, hypoxia resistance, and survival [6, 8]. Cap-independent translation is also highly correlated with drug resistance under hypoxic conditions [12].

Oxygen is necessary for cells to produce adequate amounts of ATP to support cellular metabolic activities. Hypoxic conditions are often observed within cancer tissues, due to the high proliferation rates and vascular abnormalities [13]. Hypoxia-inducible factor-1 (HIF-1), an oxygen-sensitive transcription factor, initiates adaptive responses to changes in oxygen tension in various microenvironments [14]. HIF-1 is a heterodimer protein complex composed of a constitutively expressed HIF-1β subunit and a highly regulated HIF-1α subunit [15]. The balance between protein synthesis and protein degradation determines the expression level of HIF-1α. During hypoxia, the protein synthesis process for HIF-1α mRNA switches from cap-dependent to cap-independent (IRES-dependent) translation [6]. Accumulated HIF-1α dimerizes with HIF-1β and binds to hypoxia-response elements (HREs) within the promoters of target genes, such as *VEGF*, glucose transporter 1 (*GLUT1*), erythropoietin (*EPO*), and nitric oxide synthase (*NOS*) [16]. HIF-1α overexpression and activation stimulates several key pathways in carcinogenesis, such as those related to angiogenesis, anaerobic glucose metabolism, dedifferentiation, and invasion [13].

The phenanthroquinolizidine alkaloid cryptopleurine, is a natural product isolated from the Asclepiadaceae and Moraceae families. Its anticancer activity in drug-resistant cancer cells has been elucidated [17–19]. YXM110 was shown to be the most potent cryptopleurine derivative against seven cancer cell lines. We previously reported that YXM110 inhibited global protein synthesis, and stimulated the degradation of 4E-BP1 and regulation of autophagy, which ultimately led to tumor suppression [20]. YXM110 induced decrease in 4E-BP1 expression in an *in vivo* xenograft model further confirmed its antitumor effect (unpublished data). We previously also found that under hypoxic conditions, YXM110 had the ability to reduce 4E-BP1 protein levels and compared to paclitaxel, cells showed less resistance to YXM110. Consequently, we suggested that 4E-BP1 depletion could be a benefit for an anticancer drug development.

To our knowledge, this study is the first to demonstrate that the expression of 4E-BP1 correlates with patient survival time and recurrence in CRC. Significantly increased 4E-BP1 levels were observed in all colon cancer cell lines tested as well as in the cancer tissues of patients. Clinical pathology analysis demonstrated that 4E-BP1 expression is associated with poor prognosis and cancer stage. Under hypoxia, 4E-BP1 is thought to be an important factor for cap-independent translation. Our previous study showed that YXM110 significantly suppressed the expression of 4E-BP1 [20]. Therefore, in the present study, we investigated whether YXM110 could inhibit protein synthesis under hypoxic conditions. We found that YXM110 markedly decreased 4E-BP1 expression, resulting in diminished HIF-1α-regulated VEGF expression. Taken together, our findings indicate that 4E-BP1 could be a valuable prognostic biomarker, and YXM110 appears to be a promising lead compound for CRC therapy.

## RESULTS

### 4E-BP1 is upregulated in CRC cell lines and tumor tissues

**Figure 1:**
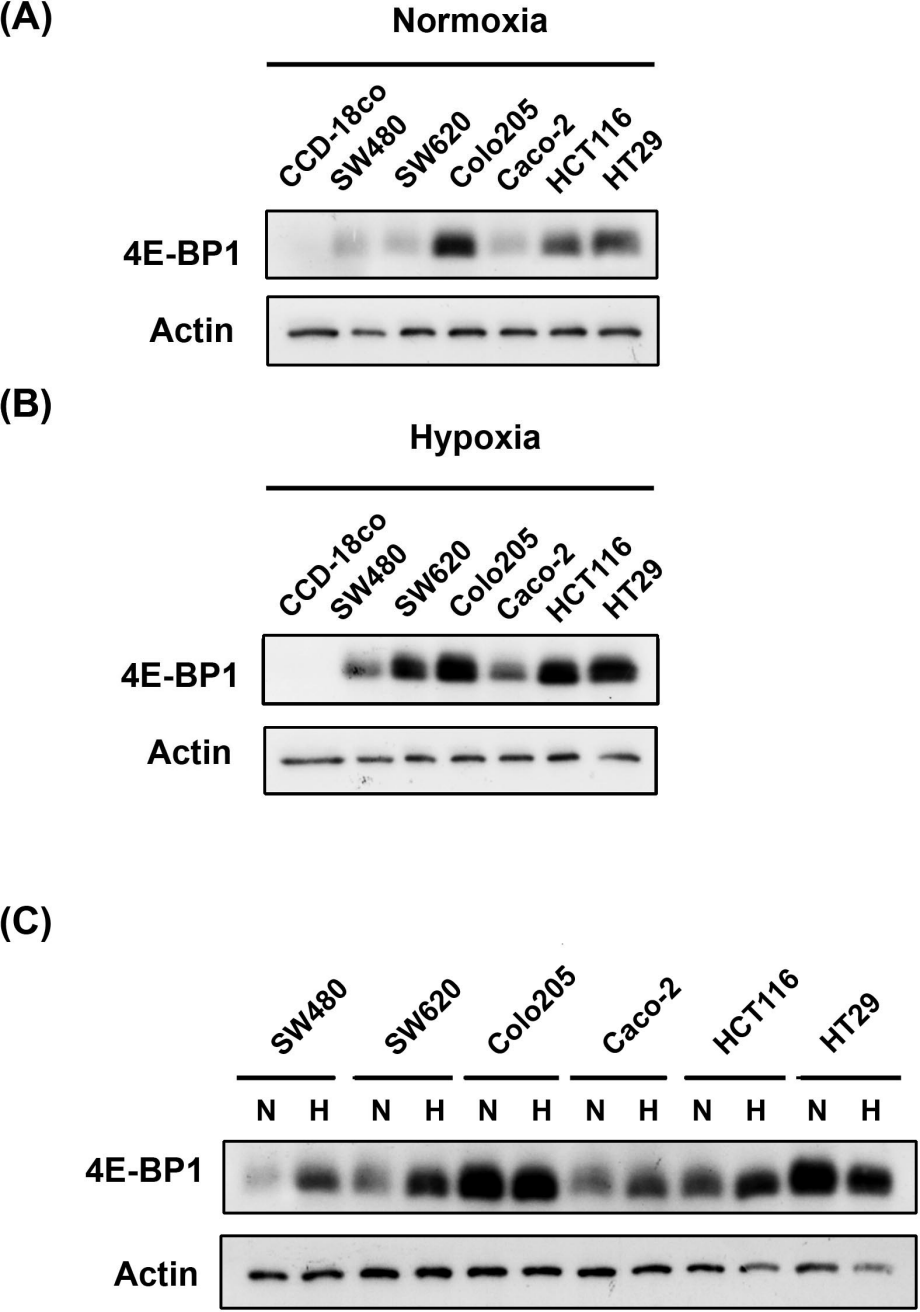
Compare 4E-BP1 expression in normal and colorectal cancer cells under normoxia and hypoxia The detection of 4E-BP1 expression in normal colon cell line (CCD-18co) and colorectal cancer cells (SW480, SW620, Colo205, Caco-2, HCT116 and HT29) under **A.** normoxia and **B.** hypoxia. **C.** Also campare the difference of 4E-BP1 between normoxia and hypoxia on each one cell line.

To elucidate the mechanistic link between 4E-BP1 and CRC, we assessed the basal levels of 4E-BP1 in 6 colon cancer cell lines (SW480, SW620, Colo205, Caco-2, HCT116, and HT-29) and 1 normal colon cell line, CCD-18co, under normoxia and hypoxia. The lowest basal protein expression levels of 4E-BP1 were observed in normal CCD-18co colon cells compared to colon cancer cell lines, under normoxic and hypoxic conditions (Figure 1A, 1B and Supplementary Figure S1A). In addition, the expression of 4E-BP1 was dramatically increased under hypoxic conditions, especially in the CRC cells, compared to its expression under normoxic conditions. This indicates that 4E-BP1 might mediate hypoxia-induced drug resistance (Figure 1C). Notably, the expression levels of 4E-BP1 in SW480, SW620, and Colo205 cells, derived from Duke's B-, C-, and D-type CRC, respectively, appeared to be elevated depending on disease progression. Caco-2 is a well-differentiated colon cancer cell line. However, 4E-BP1 levels in these cells were only slightly higher than in normal CCD-18co cells.

**Figure 2:**
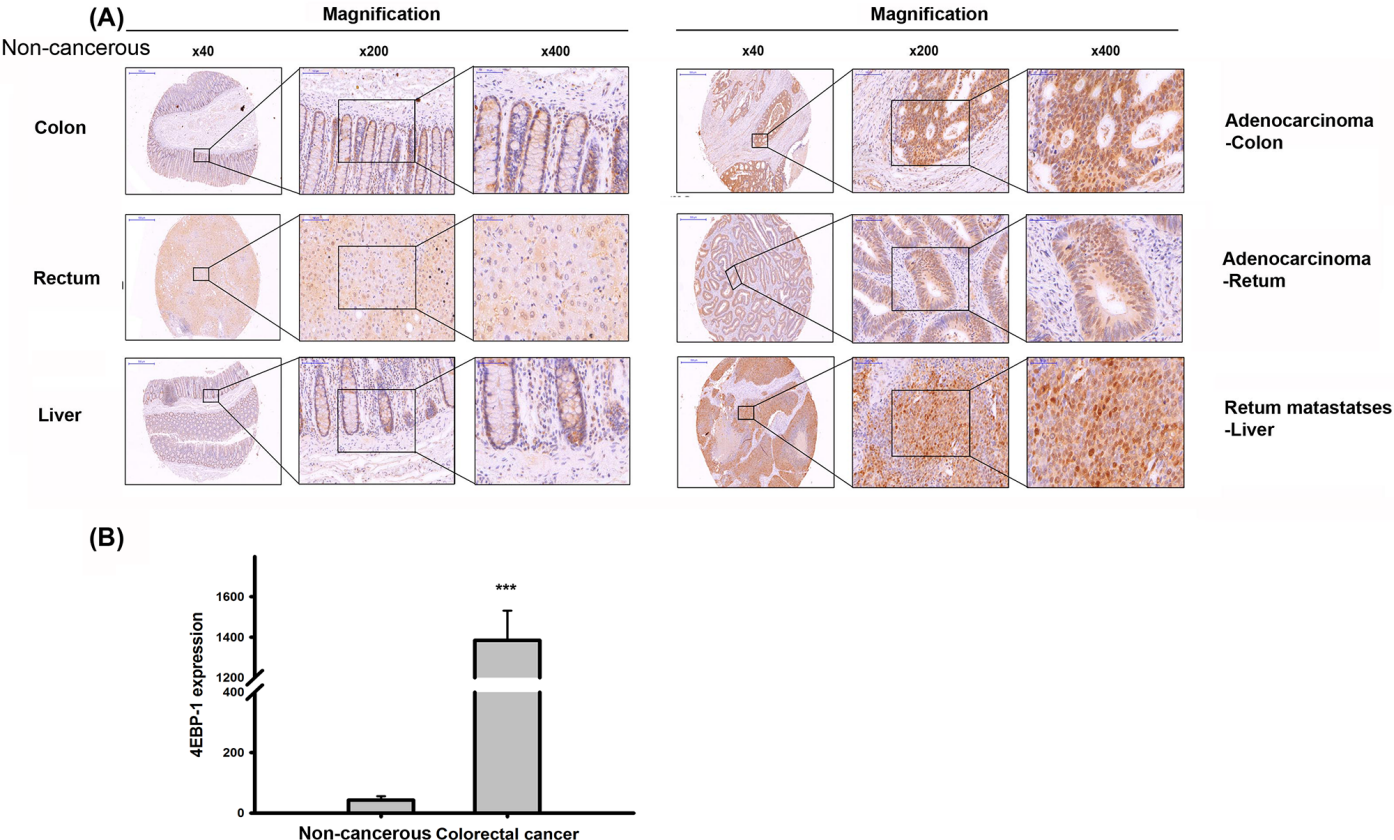
Immnuohistochemical staining of 4E-BP1 in a tissue microarray **A.** Representive 4E-BP1 staining of CRC specimens and corresponding non-cancerous tissues. **B.** The statisitc result of 4E-BP1 expression in CRC and paired normal specimens by Image J. ***P < 0.001.

4E-BP1 expression in CRC tissues was also evaluated, and 4E-BP1 was more highly expressed in cancerous colon and rectal tissue sections than in the corresponding non-cancerous sections (Figure 2A, 2B). The liver is the predominant metastatic site for CRC patients. Therefore, we also assessed 4E-BP1 expression levels in the liver in rectum metastases patients. Liver tissue from non-metastatic CRC patients expressed lower levels of 4E-BP1 than liver tissue from metastatic CRC patients. Taken together, the above results suggest that 4E-BP1 expression and tumor progression are related.

### Association of 4E-BP1 expression with clinical parameters

**Figure 3:**
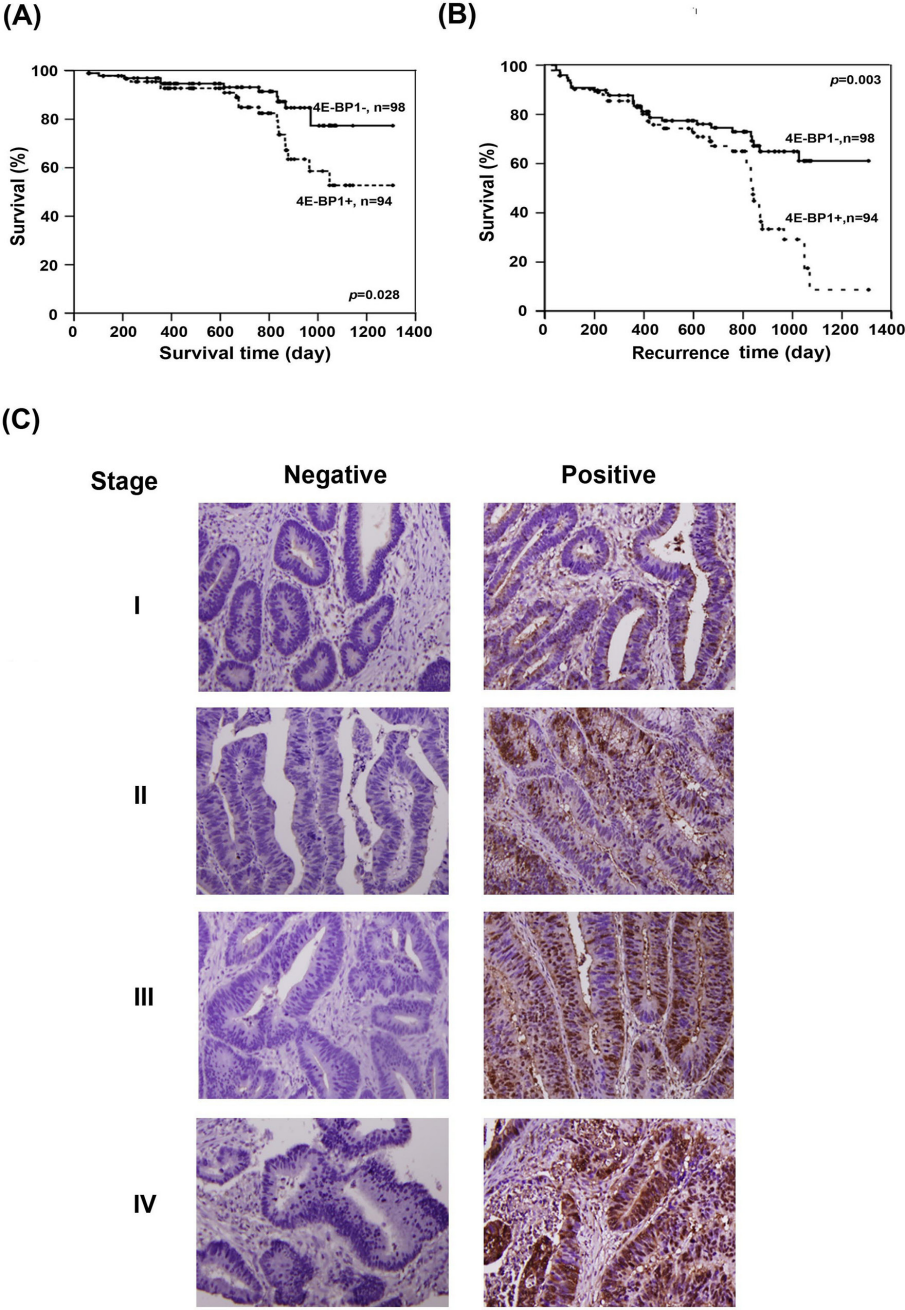
Evaluation of the clinincal importance of 4E-BP1 expression in CRC patients The correlation between 4E-BP1 and **A.** survival time and **B.** recurrence time were analyzed by Kaplan-Meier analysis. **C.** The representative of immunohistochemical staining of 4E-BP1 on different stages of sections. Left lane shows the negative staining and right lane are the positive results.

The association between 4E-BP1 and several clinical parameters, including sex, age, TNM, and cancer stage, was analyzed in 192 CRC patients (Table 1). Cancerous and non-cancerous tissues were sectioned into four parts for 4E-BP1 staining. No positive sections or only one positively stained section was defined as having “low” expression of 4E-BP1, and the presence of at least two positively stained sections was classified as “high” expression (Figure 3C). Table 1 indicates that 4E-BP1 expression in CRC patients does not correlate with age, gender, T factor (the size of the original tumor) or M factor (distant metastasis). However, we observed a significant statistical correlation with the *N* factor (lymph node invasion; *p* = 0.029) and 4E-BP1 expression. It is noteworthy that 4E-BP1 expression was more prevalent in patients diagnosed at a late stage (stages III and IV; *p* = 0.027).

**Table 1:** Association of 4E-BP1 expression and clinical parameters in tumor tissues of colorectal cancer patients

Parameters	4E-BP1		*p* value
	Low	High	
	(*n* = 98) (%)	(*n* = 94) (%)	
Age (years)			
≦65	51 (52.0)	43 (45.7)	
>65	47 (48.0)	51 (54.2)	0.391
Gender			
Female	39 (39.8)	51 (54.3)	
Male	59 (60.2)	43 (45.7)	0.060
T factor			
1	5 (5.1)	2 (2.1)	
2	15 (15.3)	13 (13.8)	
3	55 (56.1)	55 (58.5)	
4	23 (23.5)	24 (25.5)	0.713
*N* factor			
0	50 (51.0)	33 (35.1)	
1+2	48(49.0)	61 (64.9)	**0.029**
M factor			
0	83 (84.7)	76 (80.9)	
1	15 (15.3)	18 (19.1)	0.567
Stage			
I	13 (13.3)	10 (10.6)	
II	33 (33.7)	20 (21.3)	
III	36 (36.7)	46 (48.9)	
IV	16 (16.3)	18 (19.1)	0.184
Stage			
Early	46 (46.9)	29 (30.9)	
Late	52 (53.1)	65 (69.1)	**0.027**

No positive or only one positively stained section was defined as “low” expression of 4E-BP1, and the presence of at least two positively stained sections was classified as “high” expression. (*N* = 192).

### Survival analysis

The correlation between 4E-BP1 expression and clinical outcome was corroborated by a survival analysis. The patients enrolled in this study did not receive any chemotherapy before surgical resection. A Kaplan-Meier analysis showed that patients with negative results for 4E-BP1 expression exhibited longer survival times (Figure 3A) and longer time to recurrence (Figure 3B; *p* = 0.028 and 0.003, respectively). Taken together, these results indicate that the expression levels of 4E-BP1 directly correlate with CRC progression, patient survival, and recurrence. Therefore, 4E-BP1 could be a prognostic indicator and potential therapeutic target.

### YXM110 inhibits 4E-BP1 expression under hypoxic conditions and suppresses cap-independent translation via 4E-BP1 deletion

**Figure 4:**
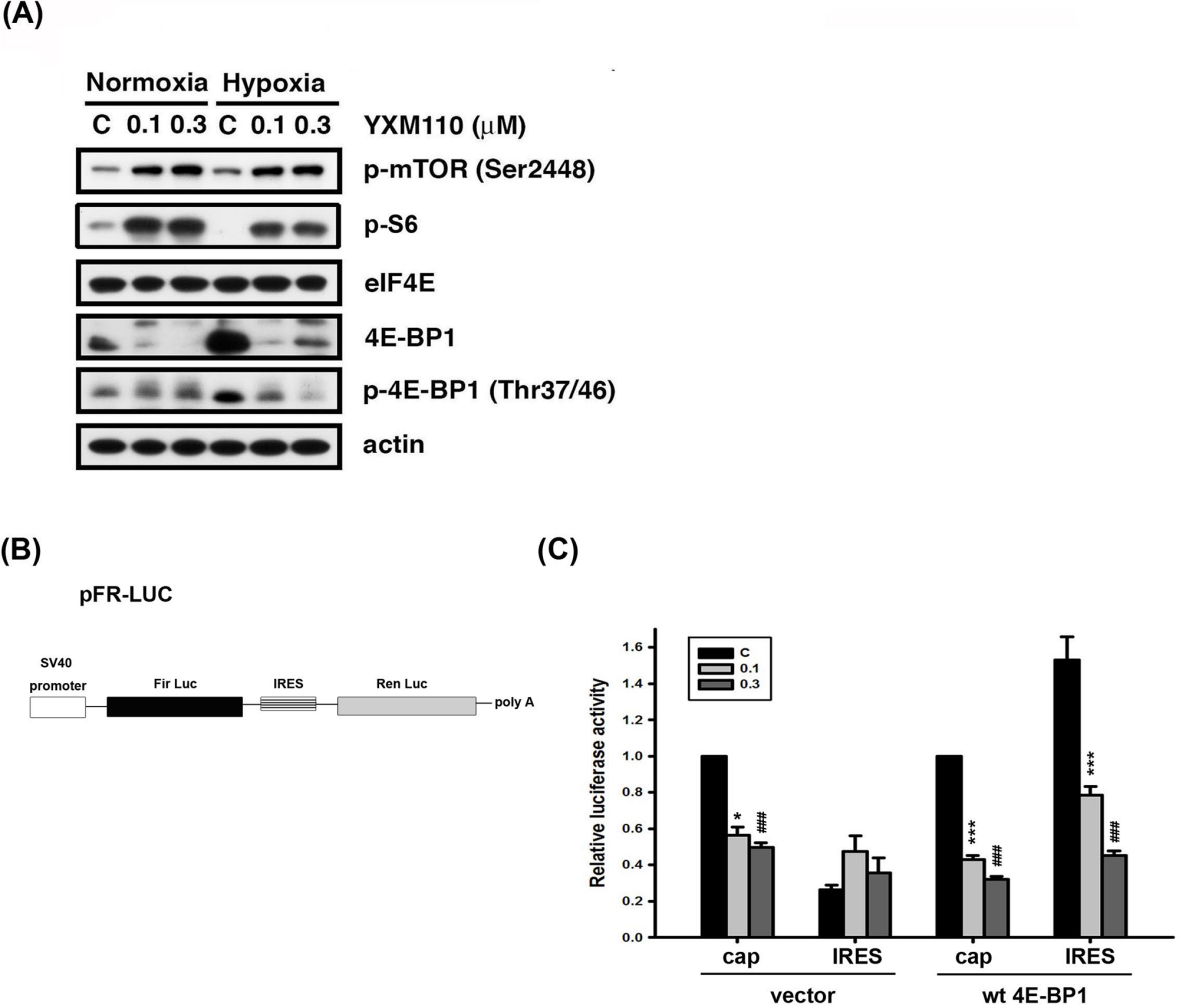
YXM110 suppresses cap-independent translation through 4E-BP1 deletion **A.** HCT116 cells were treated with vehicle (0.1% DMSO) or YXM110 (0.1 or 0.3 μM) under normoxia or hypoxia conditions for 24 h. The cells were then harvested for detection of p-mTOR (Ser2448), eIF4E, 4E-BP1, p-4E-BP1 (Thr37/46), p-S6 (Ser240/244) and actin by western blot analysis. **B.** Bicistronic luciferase vector (pFR-Luc) contains a cap-dependent firefly (Fir) and cap-independent Renilla (Ren) luciferase. **C.** HCT116 cells were cotransfected bicistronic vector and wt 4E-BP1 for testing the effect of YXM110 on cap-dependent and cap-independent translation by using a dual Luciferase reporter system. *P < 0.05; ***P < 0.001; ^###^*P* < 0.001.

Hypoxia is regarded as an obstacle to effective cancer therapy. It can cause a switch from cap-dependent to cap-independent translation [8], a process controlled mainly by 4E-BP1. As shown in Figure 1C, 4E-BP1 is upregulated in cancer cells under hypoxia, and YXM110 has been shown to inhibit its expression [20]. Therefore, we assessed the ability of YXM110 to decrease 4E-BP1 protein expression under hypoxic conditions. We observed that 4E-BP1 was significantly repressed by YXM110 under hypoxia, but this was not mediated by mTOR inhibition (Figure 4A). This implies that cap-independent translation is inhibited by YXM110. We confirmed that YXM110 suppressed cap-independent translation when 4E-BP1 was overexpressed by using a bicistronic vector harboring cap-dependent firefly luciferase and cap-independent Renilla luciferase genes (Figure 4B). Compared to paclitaxel, HCT116 cells under hypoxic conditions demonstrated increased sensitivity to YXM110 [20]. These results suggest that YXM110-induced 4E-BP1 depletion reduces the survival of cancer cells under oxygen-deprivation by making them more sensitive to anticancer agents.

### YXM110 inhibits HIF-1α translation

**Figure 5:**
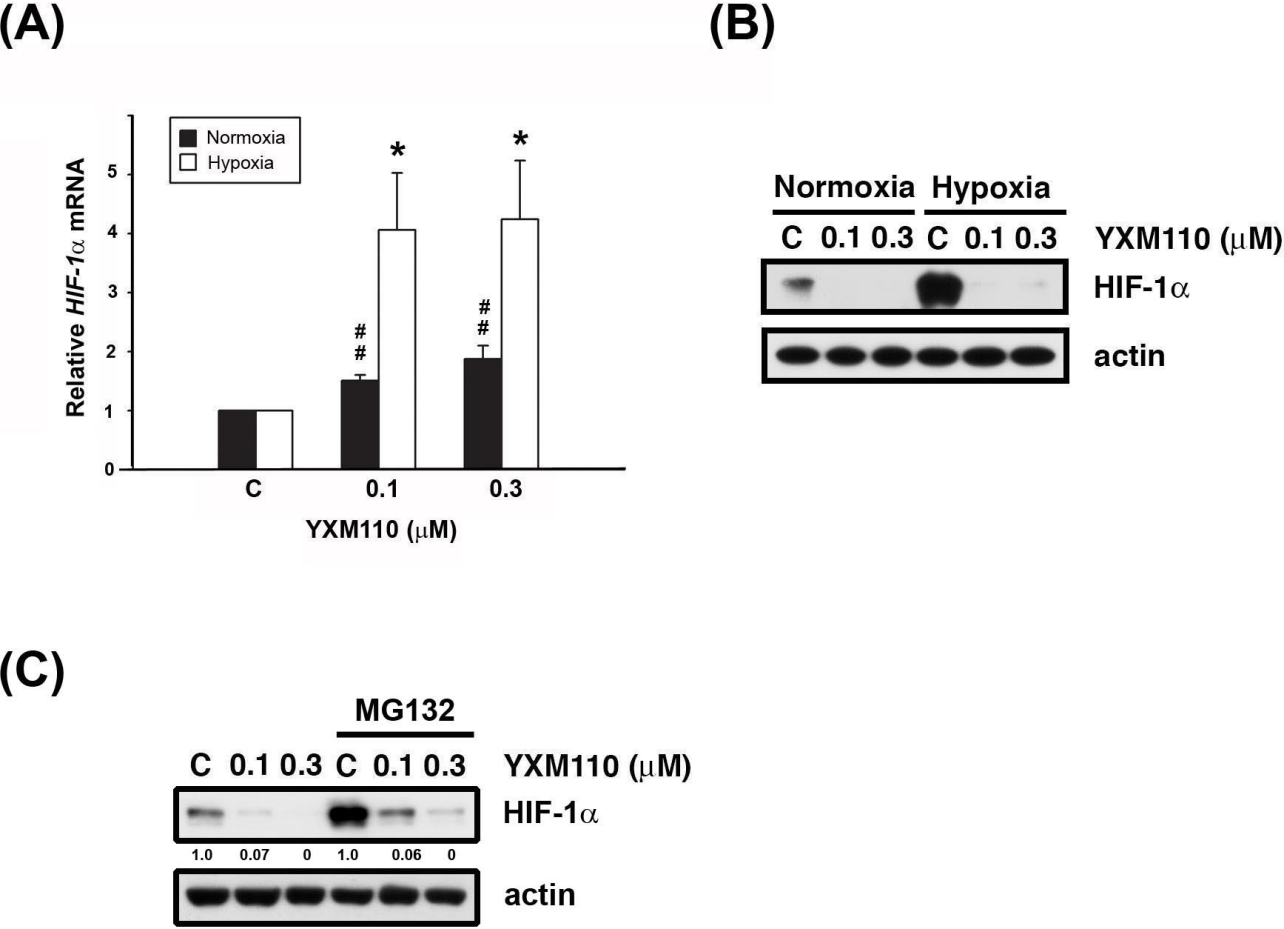
YXM110 inhibits HIF-1α translation **A.** HIF-1α mRNA was analyzed with quantitative real-time RT-PCR after 6 h treatment of YXM110 (0.1 or 0.3 μM) under normoxia or hypoxia conditions. **B.** HCT116 cells were treated with vehicle (0.1% DMSO) or YXM110 (0.1 or 0.3 μM) under normoxia or hypoxia conditions for 24 h or **C.** pretreated with vehicle or 10 μM MG132 and then incubated with vehicle or YXM110 (0.1 or 0.3 μM) for 24 h under hypoxia. The cells were then harvested for detection of HIF-1α and actin by western blot analysis. *P < 0.05; ^##^ *P* < 0.01.

The HIF-1 protein activates adaptive responses to hypoxic conditions [14]. During hypoxia, cap-dependent translation can switch to cap-independent (IRES-dependent) translation of HIF-1α mRNA [6]. Therefore, mRNA and protein levels of HIF-1α were examined after YXM110 treatment under normoxia and hypoxia. We showed that HIF-1α mRNA expression is indeed increased during hypoxia (Figure 5). However, protein expression of HIF-1α was not detectable by western blot analysis (Figure 5B), suggesting that the translation process was blocked. Pretreatment with the proteasome inhibitor, MG132 could not reverse YXM110 mediated HIF-1α downregulation (Figure 5C). However, HIF-1β protein levels were also decreased after YXM110 treatment (Supplementary Figure S2), including HIF-1β without IRES-dependent translation. There may be other mechanisms that facilitate YXM110 mediated HIF-1β downregulation. These results suggest that hypoxia-activated cap-independent translation is inhibited by YXM110.

### YXM110 inhibits HRE-luciferase activity and VEGF transcription and translation

**Figure 6:**
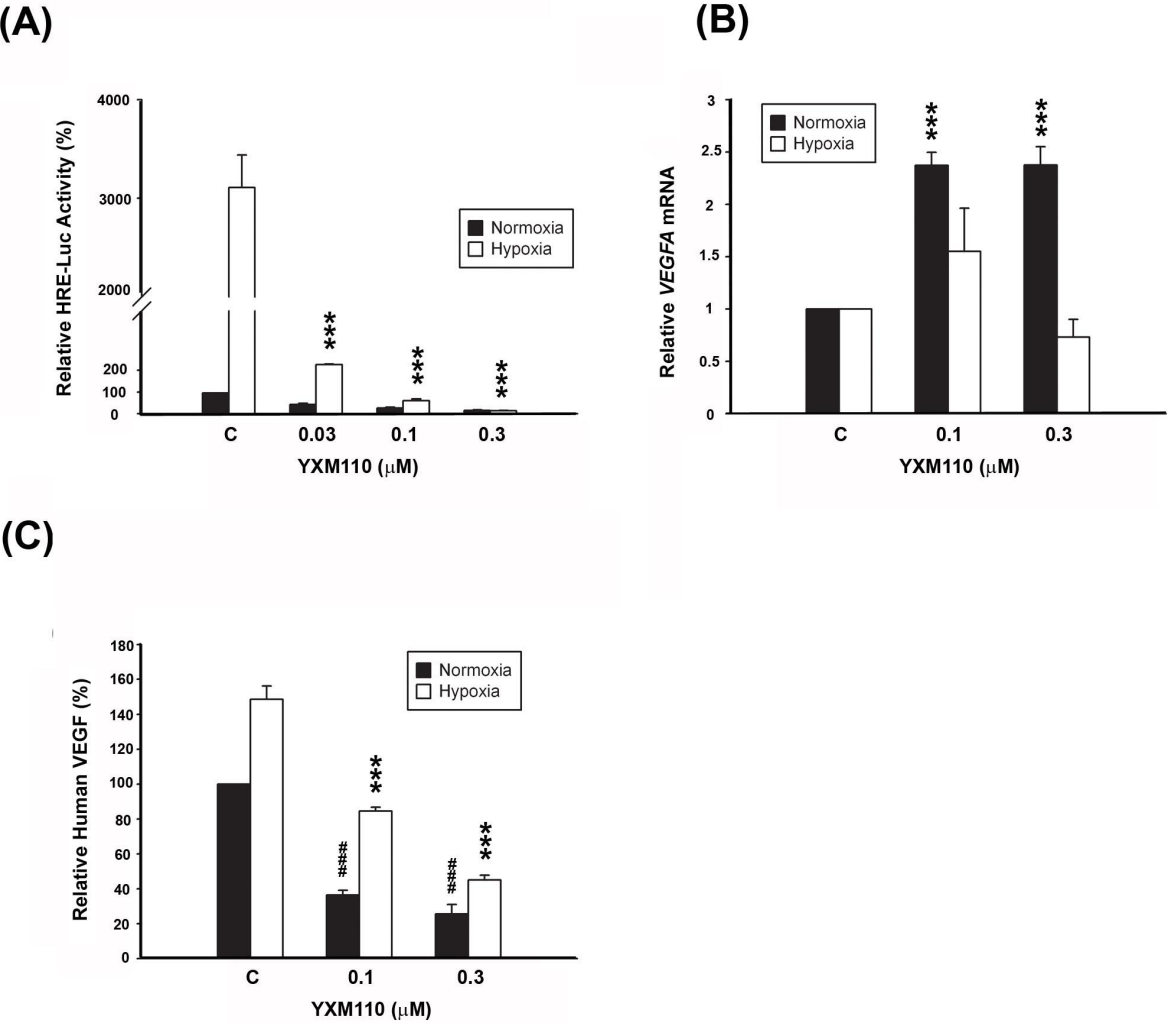
YXM110 inhibits HRE-Luciferases activity and VEGF transcription and translation **A.** HCT116 cells transiently transfected with 5-HRE-Luciferase reporter were treated with YXM110 under normoxia or hypoxia conditions for 24 h. Data have shown represented luciferase activity relative to the control. **B.** VEGFA mRNA was analyzed with quantitative real-time RT-PCR after 6 h treatment of YXM110 (0.1 or 0.3 μM) under normoxia or hypoxia conditions. **C.** VEGF levels in culture medium were determined using Human VEGF Quantikine ELISA Kit. ***P < 0.001; ^###^*P* < 0.001.

HIF-1 is a known transcription factor for dozens of hypoxia induced target genes [13–16]. HIF-1 regulates transcription at the hypoxia-responsive element (HRE) domain of the promoter. Therefore, we measured the transcriptional activity of HIF-1 via the expression of the downstream target gene *VEGF*. HCT116 cells were transfected with a reporter plasmid containing five copies of HRE with the luciferase promoter gene. The transcriptional activity of HIF-1 in hypoxic cells was approximately 3,000 fold higher than in normoxic cells. However, YXM110 effectively inhibited the hypoxia-induced transcriptional activity of HIF-1 (Figure 6A), and the transcription of hypoxia-increased VEGFA mRNA expression (Figure 6B). Furthermore, the levels of secreted VEGF decreased with YXM110 treatment (Figure 6C). Taken together, the results indicate that YXM110 inhibited HIF-1α translation and its transcriptional activity.

## DISCUSSION

The aim of this study was to demonstrate the importance of 4E-BP1 expression in CRC. All colon cancer lines evaluated exhibited higher 4E-BP1 protein levels than normal cells under normoxia and hypoxia, and a tissue microarray confirmed this result in tumors. Moreover, 4E-BP1 expression positively correlated with poor prognosis. YXM110, a new phenanthroquinolizidine, notably suppressed 4E-BP1 expression under hypoxia, with concomitant inhibition of HIF-1α-regulated VEGFA transcription and translation. Therefore, we contend that 4E-BP1 could be a valuable target for CRC treatment.

Translational regulation is crucial in cancer development and progression and can initiate tumor cell survival, angiogenesis, invasion, and metastasis. Translation of mRNAs is controlled by dual mechanisms at the level of initiation: cap-dependent and cap-independent (IRES-dependent) translation [6]. Substantial evidence has shown that global protein synthesis, which is mostly regulated by cap-dependent translation, promotes tumor cell growth and survival. In cap-dependent translation, the rate-limiting step of initiation is controlled by eIF4E, the 5′ cap-binding protein, 4E-BP1, and the eIF4E-binding protein. Therefore, the roles of the interaction and expression of eIF4E and 4E-BP1 in cancer progression has been widely examined. mTOR hyperphosphorylated 4E-BP1 is inactive and unable to bind to eIF4E in the steady state, indicating that eIF4E can initiate mRNA translation. Therefore, p-4E-BP1 is regarded as a proto-oncogene and even as a “funnel factor” in human cancers [21]. Many diverse oncogenic signals, such as Her2, Ras-Raf, and PI3K-Akt converge at 4E-BP1, affecting cancer cell proliferation, invasion, and metastasis [22]. Studies have shown that in breast cancer, p-4E-BP1 is mainly expressed in poorly differentiated tumors, and correlates with tumor size, lymph node metastasis, and recurrence [23, 24]. The ratio of 4E-BP1 to eIF4E is an indicator of rapamycin sensitivity [25, 26] in cancer cells. Increasing evidence indicates that dephosphorylation of 4E-BP1 can serve as a clinical biomarker, and mimetic compounds of 4E-BP1 have recently been developed [27].

However, cap-dependent mRNA translation is inhibited and cap-independent translation is enhanced in response to a variety of cell stressors, including growth arrest, nutrient deprivation, mitosis, hypoxia, and apoptosis [6, 11]. This suggests that cap-independent translation also plays an important role in cancer. Several studies have demonstrated that 4E-BP1 expression is elevated in human breast, prostate, head and neck, and some gastrointestinal cancers [28–30]. Furthermore, mTOR-independent 4E-BP1 expression and phosphorylation have been reported as a mechanism of primary resistance to mTOR kinase inhibitors [31–33]. Reports have shown that colon cell origin is a strong predictor for mTOR inhibitor resistance [31, 32]. In addition, 4E-BP1 overexpression is associated with poor prognosis and endocrine resistance in breast cancer [33].

As shown in Figure 1, compared to the normal colon cell line, all cancer cell lines tested had increased 4E-BP1 levels under both normoxic and hypoxic conditions. The IHC staining of patient tumors provided strong evidence that 4E-BP1 is upregulated in cancerous tissues (Figure 2). Cell line analysis demonstrated that poor progression of CRC is associated with increased 4E-BP1 protein levels. These data are corroborated by the analysis of clinical pathology parameters. Patients with lymph node metastasis had statistically significant high 4E-BP1 expression, which correlated with late-stage CRC (Table 1). This was also associated with survival and recurrence time, as patients with low 4E-BP1 expression had better clinical outcomes (Figure 3).

We also determined the expression levels of eIF4E and p-4E-BP1 in colorectal cancer cell lines and normal cells. As shown in Supplementary Figure S1B and S1D, eIF4E and p-4E-BP1 were upregulated in most colorectal cancer cells under normoxia and hypoxia. However, it is noteworthy that the expression levels of eIF4E and p-4E-BP1 were decreased in hypoxia compared to normoxia in most colorectal cells except for SW480 and SW620 cells, which suggest a role for eIF4E and p-4E-BP1 in cap-dependent translation. Moreover, the analysis of clinical CRC tumor tissues indicates that there is a positive association between 4E-BP1 and eIF4E protein expression (Supplementary Table 1), suggesting that eIF4E may have a similar role as 4EBP in CRC tumorigenesis. Since the loss of 4E-BP1 or eIF4E overexpression can promote tumorigenesis, the ratio of 4E-BP1 to eIF4E in colorectal cancer cells was also evaluated. Supplementary Figure S1C shows that the ratio of 4E-BP1 to eIF4E dramatically increased in cancer cells, especially under hypoxia.

Tumor hypoxia is considered as an obstacle to efficient cancer treatment with radiotherapy and chemotherapy because it acts as a barrier in the tumor region. Recent evidence has shown that tumor hypoxia is associated with more malignant phenotypes and poor prognosis. Therefore, developing selectively targeted therapy to hypoxic cells is an ongoing area of research [34]. In several reported studies, 4E-BP1 has been demonstrated to be a positive regulator of cap-independent mRNA translation under hypoxic conditions. Tumor hypoxia induced 4E-BP1, decreases cap-dependent translation and activates cap-independent translation of mRNAs encoding HIF1α and VEGFA, which contributes to cell survival and angiogenesis [8]. YXM110 significantly depleted 4E-BP1 expression without mTOR inhibition, decreased 4E-BP1 mediated cap-independent translation HIF-1α translation and its transcriptional activity under hypoxia (Figures 4–6). These results suggest that YXM110 represses hypoxia-activated cap-independent translation.

Based on the findings described in Table 1, we concluded that 4EBP1 expression is associated with *N* factor, which indicates that nearby (regional) lymph nodes are involved in CRC progression, as well as TNM stage. It has been reported that soluble VEGF-A was associated with lymphangiogenensis [35]. Moreover, the expression VEGF-A and VEGF-C was significantly elevated in CRCs, and tumors with lymph node metastases had significantly higher levels of VEGF-A compared with non-metastatic tumors [36, 37]. We therefore evaluated the expression levels of VEGF-A and VEGF-C after YXM110 treatment and showed that YXM110 depleted VEGF-A and VEGF-C expression at the transcriptional level (Figure 6 and Supplementary Figure S3). However, it was found that hypoxia induces VEGF-C expression via a HIF-1α-independent translation-mediated mechanism [38]. We speculated that VEGF-C translational downregulation might be due to YXM110 mediated cap-independent inhibition rather than a 4E-BP1-HIF-1αpathway.

Akt/mTOR signaling is known to regulate protein synthesis. YXM110 was proved to have an ability to inhibit protein synthesis even that YXM110 induced mTOR signaling activation (Figure 4A) [20]. It was also shown that extracellular signals activate mTOR, which in turns triggers HIF-1α induction though transcriptional regulation [39]. Although HIF-1α mRNA was induced after treatment with YXM110, HIF-1α protein was profoundly suppressed by YXM110 in a translational manner (Figure 5). Hypoxia can cause cell cycle arrest and simultaneously suppresses conversion from proliferative arrest to cellular senescence [40–41]. Nonetheless, inhibition of 4E-BP1 can sensitize cells, which is hypoxia tolerance and radioresistance, to chemotherapy and irradiation [42]. We recently reported that compared to anticancer drugs, YXM110 can reduce the survival of cancer cells during hypoxia [20]. These combined observations strongly suggest that YXM110, an inhibitor of 4E-BP1, may be an effective agent for treating drug-resistant CRC.

In conclusion, to the best of our knowledge, this study is the first to demonstrate the significance of 4E-BP1 in CRC through clinical data. Although 4E-BP1 expression is paradoxically elevated in some tumors and can act as a tumor suppressor or proto-oncogene, we emphasize that, in this study, the expression of 4E-BP1 is associated with poor prognosis in CRC. Here we provide a novel mechanism of action for the cryptopleurine derivative, YXM110. We show that it significantly depletes 4E-BP1 expression and subsequently induces the suppression of cap-independent translation with the inhibition of HIF-1α-regulated VEGFA transcription under hypoxia, which facilitates drug resistance. Therefore, from a therapeutic perspective, 4E-BP1 could be a promising prognostic marker and an excellent drug target for treatment of CRC. Accordingly, YXM110 is a potential lead therapeutic compound, particularly for treatment of drug-resistant CRC.

## MATERIALS AND METHODS

### Study subjects

Primary colorectal cancer patients were admitted to Taipei Medical University Hospital, China Medical University Hospital and Chung Shan Medical University Hospital. All of them wrote informed consent approved by the Institutional Review Board (IRB number: 201402018 and 201312018). The enrolled patients did not receive any chemotherapy or radiation therapy prior to surgery. The TNM stages of all colorectal cancer patients were determined according to the American Joint Committee on Cancer/International Union Against Cancer TNM staging system.

### Immunohistochemical staining for 4E-BP1 protein expression

Non-cancerous and colorectal cancer tissues were sectioned from patients and formalin-fixed and paraffin-embedded. These sections were deparaffinized with xylen, rehydrated in different concentration of alcohol and immersed in 3% H_2_0_2_ for removing endogenous peroxidase. Sections were heated in a microwave in citrate buffer (pH 6.0) for antigen retrieval. Washing with PBS, the sections were blocked with 3% BSA/PBS for 30 min and incubated with anti-4E-BP1 antibody (Cell Signaling) at 4°C overnight. The streptavidin peroxidase method was performed (Dako, LSAB kit) to develop the signal.

### Cell culture and hypoxia

Human colorectal cancer cell line HCT116, HT-29 and Colo205 were purchased from American Type Culture Collection (ATCC; Manassas, VA, USA). SW480, SW620, Caco-2 and CCD-18co were kindly gift from Min-Chuan Huang (Graduate institute of Anatomy and Cell Biology, College of Medicine, National Taiwan University) and Kuen-Haur Lee (Program for Cancer Biology and Drug Discovery, College of Medical Science and Technology, Taipei Medical University). HCT116, HT-29 and Colo205 were cultured in RPMI-1640 medium (Gibco, Invitrogen, Carlsbad, CA, USA); SW480, SW620 and Caco-2 were maintained in DMEM medium (Gibco, Invitrogen, Carlsbad, CA, USA); CCD-18co was in MEM medium (Gibco, Invitrogen, Carlsbad, CA, USA). All of them were supplemented with 10% heat-inactivated FBS, penicillin 100 U/ml, streptomycin 100 μg/ml, and amphotericin B 2.5 μg/ml. Cells were maintained at 37°C in a humidified incubator with 5% CO_2_/95% air. Hypoxia incubations were performed in hypoxia chamber, which was flushed with 1% O_2_, 5% CO_2_, and 94% N_2_ at 37°C (BioSpherix Proox 110), and incubated for the indicated time.

### Western blot analysis

For total lysate, cells were lysed with the 120 μL ice-cold lysis buffer [10 mmol/L Tris-HCl (pH 7.4), 150 mmol/L NaCl, 1 mmol/L EGTA, 1 mmol/L phenylmethylsulfonyl fluoride, 10 μg/mL aprotinin, 10 μg/mL leupeptin, 1 mM sodium orthovandate, 1 mM NaF, and 1% Triton X-100]. Cell lysates were centrifuged at 13, 000 rpm for 30 min. For Western blot analysis, the amount of protein (40–60 μg) was separated by electrophoresis in 10%-15% polyacrylamide gels and transferred to polyvinylidene difluoride (PVDF) membranes. After 1 h incubation at room temperature in PBS/5% nonfat milk, the membrane was washed with PBS/0.1% Tween 20 and incubated with the indicated antibodies at 4°C overnight. After washing with PBS/0.1% Tween 20, the corresponding secondary antibodies were applied to the membranes for 1 h at room temperature. The membranes were then washed with PBS/0.1% Tween 20 and the detection of signal was done with an enhanced chemiluminescence detection kit.

### Quantitative RT-PCR

Total RNA was extracted with TRIzol reagent by the manufacturer's protocol (Invitrogen, USA). 5 μg mRNA was incubated with random primer at 65°C for 5 min, then mixed with M-MLV RT at 37°C for 1 h to obtain cDNA. The SYBR Green PCR reaction (SYBR^®^ Green PCR Master Mix; Roche, Switzerland) was used to evaluate the amplification of *HIF-1α* and *VEGFA* genes. Primer sequences used for amplification are as follows: HIF-1α, 5′-ATC CAT GTG ACC ATG AGG AAA TG-3′ and 3′-TCG GCT AGT TAG GGT ACA CTT C-5′; HIF-1β, 5′-TTTATCCCTAGAGATGGGTACAGG-3′ and 5′-CCACAGGCTGGACAGAAACC-3; VEGFA, 5′-AAC CAT GAG TTT ATC GCC ACC-3′ and 5′-AGC GTT ACA TTG CCT GCA TTT-3′; VEGF-C, 5′-GCCAACCTCAACTCAAGGAC-3′ and 5′-CCCACA TCTGTAGACGGACA-3′. Fluorescent signal was detected and recorded by StepOne Real-Time PCR System (Applied Biosystems, USA), and each amplification reaction was checked for the absence of nonspecific PCR products by melting curve. Relative fold changes in gene expression are calculated as 2^ΔΔCt^.

### Transient transfection

For luciferase assay, cells were seeded in 24-well plates (15,000 cells/well) overnight, and then transfected with 5-HRE-Luciferase reporter plasmid with Lipofectamine 2000 Transfection Reagent (Invitrogen) according to the manufacturer's protocol. HCT116 cells were treated with vehicle (0.1% DMSO) or YXM110 (0.1 or 0.3 μM) under normoxia or hypoxia conditions for 24 h. The promoter activity was determined by using Luciferase Assay System (#E1500) from Promega. For cap-dependent and cap-independent translation assay, cells overexpressing wild-type 4E-BP1 were transfected with pFR-LUCs plasmid, which is a kind gift from Tarn, Woan-Yuh (Institutive of Biomedical Sciences, Academia Sinica, Taiwan), expressing bicistronic mRNA reporter containing a cap-dependent firefly luciferase and Renilla luciferase. The dual luciferase activity was determined by using Dual-Luciferase^®^ Reporter Assay (Promega).

### VEGF ELISA

HCT116 cells were seeded in 24-well (15,000 cells/well) overnight, and then treated with vehicle or YXM110 under normoxia or hypoxia conditions for 24 h. Culture medium were collected to measure the amount of VEGF by Human VEGF Quantikine ELISA Kit (R&D, Minneapolis, USA) according to the manufacturer's instructions.

### Statistical analysis

Logistic regression analysis was used to assess the association of 4E-BP1 and age, gender, tumor progression. Survival and recurrence time were estimated by Kaplan-Meier method. And data were expressed as mean ± SEM of the indicated number for separate experiments. Statistical analysis of data was performed with one-way ANOVA followed by the Student's *t* test, and *p* < 0.05 were considered significant (*P < 0.05, **P < 0.01, ***P < 0.001).

## SUPPLEMENTARY FIGURES AND TABLE


