# Supplementary material for: eIF4E binding protein 1 expression is associated with clinical survival outcomes in colorectal cancer
Source: Oncotarget. 2015 Jun 29;6(27):24092–104. doi: 10.18632/oncotarget.4483 (PMC4695172; doi:10.18632/oncotarget.4483)
Supplement: Supplementary file 1 [file oncotarget-06-24092-s001.pdf]

SUPPLEMENTARY FIGURES AND TABLE

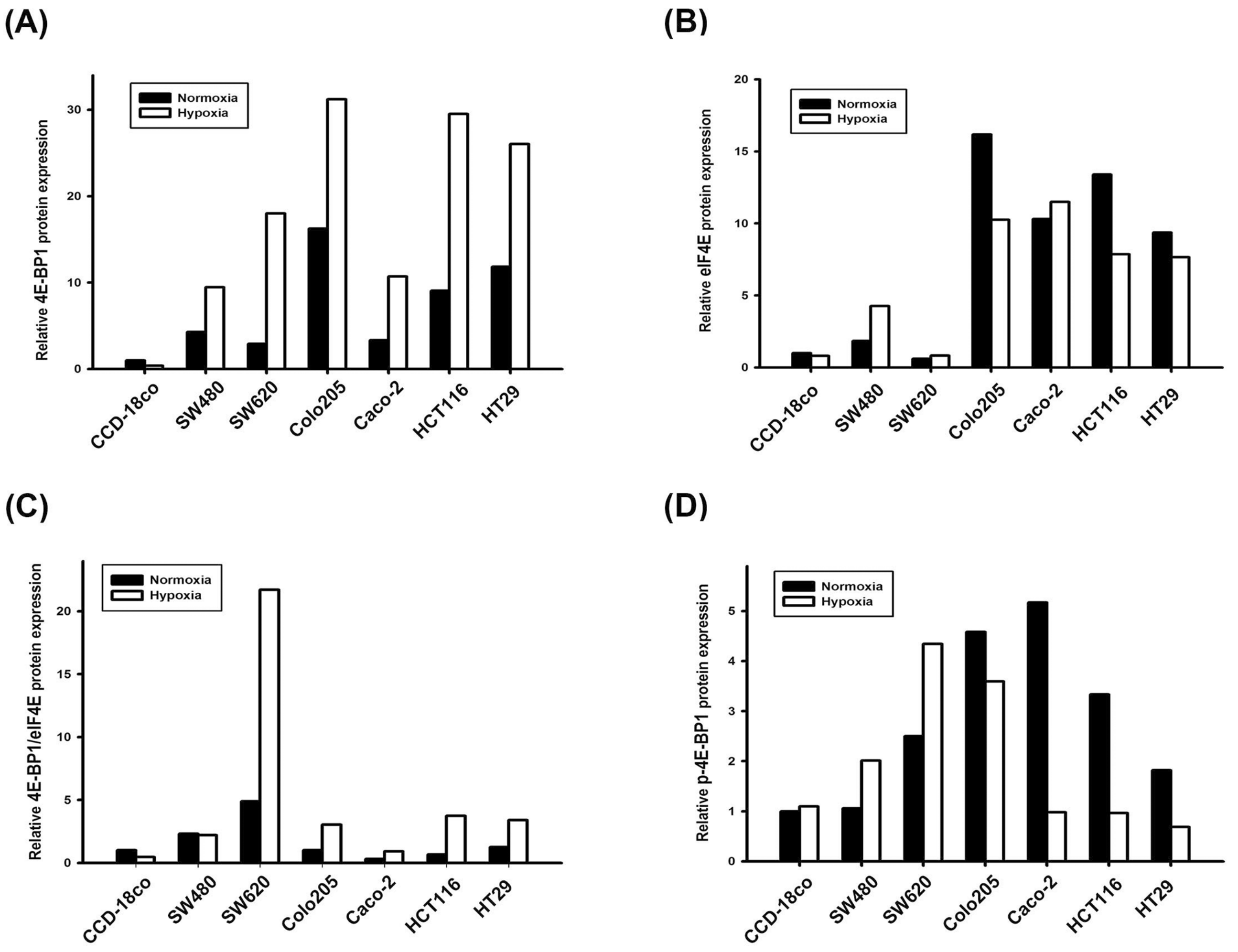

Supplementary Figure S1: Compare basal expression of 4E-BP1, eIF4E and p-4E-BP1 in normal colon cell line and colorectal cancer cells under normoxia and hypoxia. The quantitative results of **A.** 4E-BP1, **B.** eIF4E, and **D.** p-4E-BP1 expression and **C.** the ratio of 4E-BP1:eIF4E in normal colon cell line and colorectal cancer cells under normoxia and hypoxia.

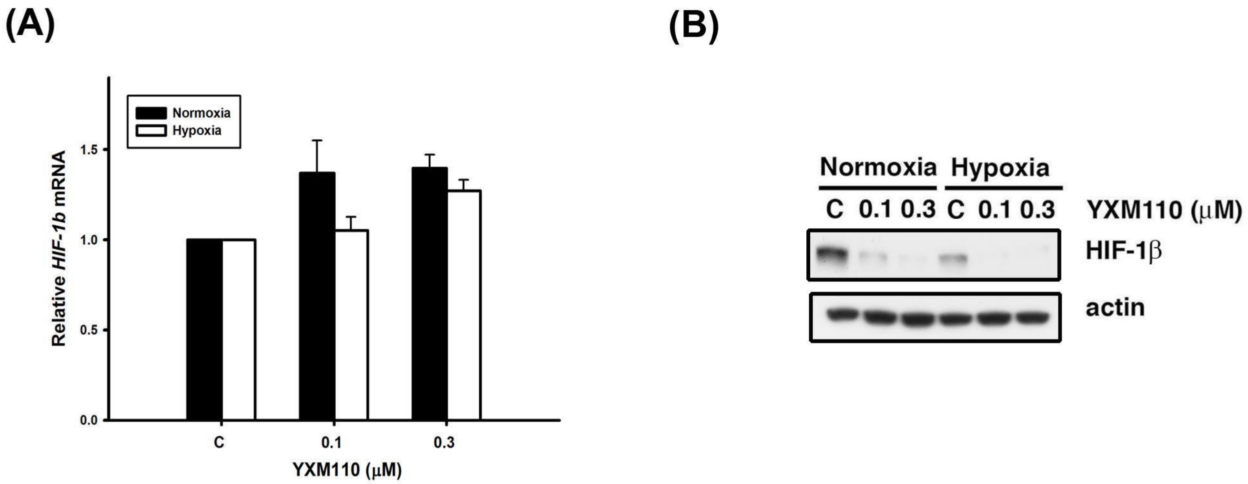

Supplementary Figure S2: YXM110 inhibits HIF-1β protein levels. A. HIF-1β mRNA was analyzed with quantitative real-time RT-PCR after 6 h treatment of YXM110 (0.1 or 0.3 μM) under normoxia or hypoxia conditions. **B.** HCT116 cells were treated with vehicle (0.1% DMSO) or YXM110 (0.1 or 0.3 μM) under normoxia or hypoxia conditions for 24 h. The cells were then harvested for detection of HIF-1β and actin by western blot analysis.

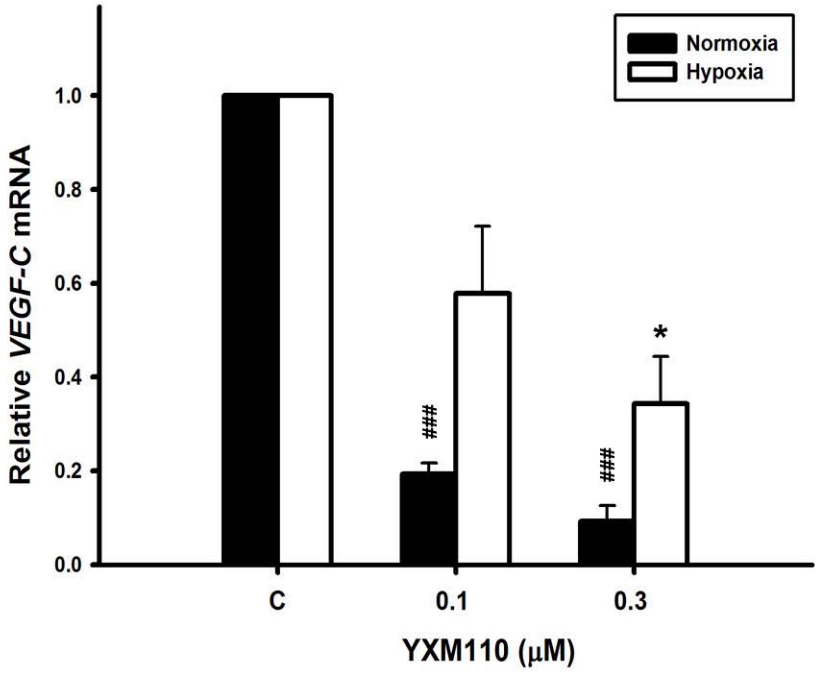

Supplementary Figure S3: YXM110 suppresses VEGF-C transcriptional level. VEGF-C mRNA was analyzed with quantitative real-time RT-PCR after 6 h treatment of YXM110 (0.1 or 0.3 μM) under normoxia or hypoxia conditions.. $^{*}P < 0.01$; $^{###}P < 0.001$.

Supplementary Table S1: The correlation of 4EBP1 and eIF4E expression in tumor tissues of CRC

	4EBP1		
	**Low ($n = 30$)**	**High ($n = 30$)**	***p* value**
eIF4E			
Low ($n = 35$)	25	10	
High ($n = 25$)	5	20	<0.0001

No positive or only one positively stained section was defined as "low" expression of 4E-BP1 or eIF4E, and the presence of at least two positively stained sections was classified as "high" expression. ($N = 60$).
